# Unilateral Jumping the Bite

**Published:** 1901-05

**Authors:** Eugene S. Talbot

**Affiliations:** Chicago


### UNILATERAL JUMPING THE BITE.

BY EUGENE S. TALBOT, M.D., D.D.S., CHICAGO.

A THIRTY-TWO-YEAR-OLD woman came to Chicago from a Western State to obtain relief from constant pain and fatigue. She is a degenerate. She has been married nine years and has one child. She has been losing flesh for a number of years, and has rheumatism in her limbs. There is constant pain in the occipital region and in the right glenoid fossa. The appetite is poor, assimilation defective, and she is badly nourished. She had considerable neuralgia. Her family physician and dentist being unable to relieve her, she was sent to a Chicago neurologist, who found the pain most intense on the right side of the face and neck. She was then referred to me.

The lower jaw is arrested. The lower incisors meet in a line drawn across the first bicuspids. The superior incisors and jaw extend half an inch beyond the inferior. When the mouth is closed the upper lip rests upon the superior alveolar process, while the lower curls underneath, continually exposing the superior incisors.

The face describes nearly a straight line from the neck to the tip of the nose. Ten years ago she had the lower right and left first molars, upper right cuspid, first and second bicuspids, and left first molar extracted. The result was that the teeth immediately changed positions. When I saw her the teeth had so moved about that the posterior edge of the left upper and lower second molar, the left lower cuspid, and the left upper first bicuspid just touched. Upon the right side the second lower bicuspid, upper first molar, and two second molars touch. The spaces between these teeth rendered mastication impossible. The lower jaw did not rest easily. The palatine cusp on the right superior second molar and the posterior lingual cusp in the inferior second molar were degenerate teeth and exceedingly prominent, so that when the jaw opened and closed the two cusps acted as an inclined plane, throwing the right condyle out of the socket one-fourth of an inch. This dislocation extended over a period of nine years. This was the cause of the pain, tired and exhausted feeling. Improper mastication of food caused the malassimilation. She could not masticate her meat, hence she did not eat it.

The effect of forward movement of the lower jaw, or "jumping the bite" (as Kingsley terms it), is to draw the condyle forward out of the socket. This places the capsular and stylo-maxillary ligaments on a continual stretch. The condyle rests upon an inclined plane, the eminentia articular and tubercle. An effort to hold the jaw in this position can be accomplished only when the person is conscious, since the movement is voluntary. During sleep, the jaws naturally return to a restful position. In the case of the patient under discussion, owing to loss of teeth, there was only one position in which the jaws could meet. The prominent cusps of the superior and inferior molars passed each other, throwing the condyle out of its socket and firmly locking the jaws. The continual stretching of the ligament caused not only pain, but weariness and exhaustion. The cusps were ground away, the four anterior teeth removed. A gold crown was placed upon the lower molar to fill the space and bring the teeth on a line. A plate was fitted to the upper jaw, and proper articulation was secured throughout the entire dental arch. The condyle remained in its proper position. She could chew beefsteak with a relish before she left Chicago. She experienced an uncomfortable feeling at the joint owing to the abnormal condition of the ligaments. The pain, however, ceased. Her face (which had a careworn look) brightened. She continued to improve markedly in health after her return home.

